# How labour intensive is a doctor-based delivery model for antiretroviral treatment (ART)? Evidence from an observational study in Siem Reap, Cambodia

**DOI:** 10.1186/1478-4491-5-12

**Published:** 2007-05-01

**Authors:** Wim Van Damme, Soy Ty Kheang, Bart Janssens, Katharina Kober

**Affiliations:** 1 Department of Public Health, Institute of Tropical Medicine, Antwerp, Belgium; 2 Médecins Sans Frontières – Belgium, Phnom Penh, Cambodia

## Abstract

**Background:**

Funding for scaling-up antiretroviral treatment (ART) in low-income countries has increased substantially, but the lack of human resources for health (HRH) is increasingly being identified as an important constraint for scaling-up ART.

**Methods:**

In a clinic run by Médecins Sans Frontières in Siem Reap, Cambodia, we documented the use of doctor-time for ART in September 2004 and in August 2005, for different phases in ART (pre-ART, ART initiation, ART follow-up Year 1, & ART follow-up Year 2). Based on these observations and using a variety of assumptions for survival of patients on ART (between 90 and 95% annually) and for further reductions in doctor-time per patient (between 0 and 10% annually), we estimated the need for doctors for the period 2004 till 2013 in the Siem Reap clinic, and in a hypothetical district in sub-Saharan Africa.

**Results:**

In the Siem Reap clinic, we found that from 2004 to 2005 the doctor-time needed per patient was reduced by between 14% and 33%, thanks to a reduction in number of visits per patient and shorter consultation times. In 2004, 2.06 full-time equivalent (FTE) doctors were needed for 522 patients on ART, and in 2005 this was slightly reduced to 1.97 FTE doctors for 911 patients on ART. By 2013, Siem Reap clinic will need between 2 and 5 FTE doctors for ART. In a district in sub-Saharan Africa with 200,000 inhabitants and 20% adult HIV prevalence, using a similar doctor-based ART delivery model, between 4 and 11 FTE doctors would be needed to cover 50% of ART needs.

**Conclusion:**

ART is labour intensive. Important reductions in doctor-time per patient can be realized during scaling-up. The doctor-based ART delivery model analysed seems adequate for Cambodia. However, for many districts in sub-Saharan Africa a doctor-based ART delivery model may be incompatible with their HRH constraints.

## Background

Globally there are some 42 million people living with HIV/AIDS (PLWHAs), most of them in low-income countries. Cambodia is the country with the highest HIV prevalence in Asia. In 2003, adult HIV prevalence in Cambodia was assessed as 1.9%, with an estimated 123 000 PLWHAs. This is a significant decrease from 1997, when HIV prevalence was 3.0%.

Over recent years the prices of antiretrovirals (ARV) have dropped dramatically and pilot projects have proved that treating AIDS in the poorest regions of the world is feasible, with clinical outcomes, such as adherence, evolution of CD4 counts (which indicate the strength of an immune system and how far the disease has advanced) and mortality, being similar to those obtained in resource-rich settings [1,2]. International attention has focussed increasingly on the expansion of access to anti-retroviral treatment (ART) for PLWHAs. WHO's '3 by 5' initiative described the expansion of ART to millions of people as a "global health emergency", and the G8 declared now to aim at "Universal Access to ART" by 2010 [3]. Thanks to major new international initiatives, such as the Global Fund, the World Bank's Multi-country AIDS Programme (MAP) and the US. President's Emergency Plan for AIDS Relief (PEPFAR), total funding for ART has increased substantially. Most countries with a high HIV/AIDS burden do no longer lack the funds for initiating and expanding ART programmes. However, it is becoming clear that presently the main bottleneck for scaling-up ART is the absorptive capacity of health systems. In particular, the lack of human resources for health (HRH) is increasingly being identified as the main constraint [4]. Only a few reports have analysed how labour intensive ART for AIDS patients is [5]. Furthermore, such reports were based entirely on one-time observations in clinics delivering ART, usually when such clinics were in their early stages, with staff still being relatively inexperienced. It is on this basis that extrapolations have been made of the human resources needs for ART scale-up [6,7].

In this study we focus on the use of medical doctor time for ART delivery in a chronic diseases clinic in Siem Reap, Cambodia, and how this evolved over one year. We then extrapolate the findings over the 10-year period 2004–2013, for the Siem Reap clinic, and for a district-wide expansion of ART to a hypothetical district in sub-Saharan Africa.

## Methods

### Setting: the chronic diseases clinic in Siem Reap

The clinic was set up in March 2002 by the humanitarian medical organization Médecins Sans Frontières as an ambulatory care centre for adult patients with chronic conditions in the compound of the provincial hospital of Siem Reap. The principal chronic diseases treated at the clinic are, in descending order of importance: HIV/AIDS, diabetes and hypertension. In October 2002, the first patients were started on ART. Voluntary counselling and testing (VCT) is provided in a health centre of the Ministry of Health (MoH). The clinic has relations with a network of NGOs and peer support groups for PLWHAs who organise social services and home-based care. Technical support services such as laboratory, X-rays and ultrasound are provided by the provincial hospital. Samples for CD4 counts are sent to the Pasteur Institute in Phnom Penh. An NGO-run hospital in Siem Reap town treats children with AIDS.

The clinic prescribes ART for all HIV-positive patients with a CD4 count below 200. Its ART protocol uses as the standard first line treatment a fixed-dose combination consisting of lamivudine (3TC), stavudine (d4T) and nevirapine. Zidovudine and efavirenz are alternative first-line when the patient develops side-effects, drug interaction or toxicities. The clinic uses tenofovir, lamivudine (3TC) and lopinavir-boosted ritonavir (Kaletra^®^) for those who are failing to respond to the first line regimen. Before ART initiation, haemoglobin and the liver enzyme ALT are measured at week 2 and month 1. CD4 is measured every 6 months for all HIV-positive patients.

In Cambodia, the overall lack of medical personnel is not a constraining factor for ART; although misdistribution of staff may create local shortages. Doctors are trained for 6 six years at the University of Phnom Penh, using a curriculum largely inspired by French medical schools.

### Use of doctor-time for ART in Siem Reap clinic, 2004 and 2005

In September 2004 and again in August 2005, we assessed the use of doctor-time for HIV positive patients on ART. Our main focus was on the length and frequency of medical doctor consultations for HIV positive patients, from their first day consultation at the clinic to their latest ART follow-up visit. Data on the length of patient-doctor encounters was obtained by direct observation of 12 consultations during one day, followed by discussions with the doctors. Data on the frequency of doctor-patient encounters was obtained from the clinic's database. In September 2004, the clinic had few patients in follow-up for more than one year. Our second visit at the end of August 2005 allowed us to observe changes in the frequency and length of doctor-patient encounters as well as document and compare the time doctors were spending on the follow-up of patients in year one and year two and beyond.

**Figure 1:**
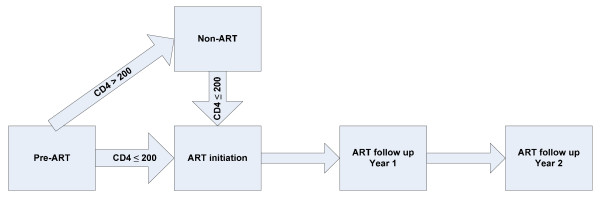
Phases in ART.

We structured our analysis by grouping HIV positive patients in subsequent phases which we labelled 'preART', 'non-ART', 'ART initiation', 'ART follow-up year 1', and 'ART follow-up year 2' (Figure 1).

The 'pre-ART' phase includes the registration of new patients, CD4 count and management of the patient. When a CD4 count reaches less than 200/μl, baseline laboratory parameters are taken, and the patient is further assessed till the decision is taken to start ART.

When a CD4 count is above 200, patients are seen periodically as 'non-ART', including 6-monthly CD4 measurements, until CD4 drops below 200. Following this, the patients go through 'ART initiation'.

The 'ART-initiation' phase includes three sessions of ART counselling and an assessment of the ART regimen best suited for the patient.

'ART follow-up year 1', includes the periodic, often monthly, consultations for patients on ART during the first year.

'ART follow-up year 2' covers the periodic visits to the clinic in the second year. This phase can be further extended into year 3, year 4 and, if required, into subsequent years.

From the data on length and frequency of doctor-patient encounters in September 2004 and in August 2005, we estimated the reduction in use of doctor-time over the course of one year.

### Use of doctor time in Siem Reap clinic, simulation 2004 – 2013

Starting from the observations made, we then used a simple extrapolation to project the expected doctor-time needed for medical consultations over the ten years between 1 January 2004 and 31 December 2013. We assumed that the Siem Reap clinic would have started on 1 January 2004, and have put 40 new patients per month on ART – as they did in 2004 and 2005 – and this until the end of 2013. For 2004 and 2005, we used the doctor time per patient, as observed. For 2006 through to 2013, we made a combination of assumptions regarding survival of patients, and possible further reduction in doctor time per patient.

For survival of patients and reduction of doctor-time per patient, we did a simulation exercise based on extrapolations with two variables:

1. survival of patients on ART, all stages of the disease included, is

a. either 90% survival per year, or

b. 95% survival per year; and

2. doctor-time per patient on ART,

a. remains the same after 2005, with no further reduction: '0% doctor-time reduction'; or

b. there is a continued annual 5% reduction of doctor-time per patient every year: '5% doctor-time reduction'; or

c. there is a continued annual 10% reduction per year, every year: '10% doctor-time reduction'.

We assume that all deaths occur on the last day of every year, either 10% or 5% of PLWHAs on ART. This is a simplification that will tend to overestimate the time needed for patients who die early in the year, but compensates somehow for the often intensive follow-up of patients over the weeks and months before their death.

We assumed that doctor-time needed for HIV positive patients not yet needing ART will remain constant over the years, and that during the first year of the simulation all patients needed a full year-equivalent of follow-up time, even those who started later in the year, and were thus followed up on for less than a full year. This assumption may somehow overestimate the need for doctor-time in the first year, but it is consistent with the observation that in the beginning, due to inexperience, doctors often conduct very long consultations with patients on ART. We estimated that a doctor sees patients for an average of 125 hours per month, or 1500 hours per year, and consequently used these estimates to calculate monthly or yearly full-time equivalents (FTE).

### Extrapolations district-wide, 2004–2013

With similar assumptions, we estimated with simple extrapolations the need for doctors to treat adults with ART in a hypothetical district that resembles districts in high burden countries of central and Southern Africa, with 200,000 inhabitants, 50% of whom are adults (= 100 000 adults), with a HIV prevalence of 20% (= 20 000 HIV positive adults). We estimated the annual need for new ART to be 10% of all HIV positive adults (= 2000 per year), and that the health services would manage to put 50% of those in need on ART (= 1000 new ART per year). We used a simulation exercise, similar to the one for the Siem Reap clinic, with the same variables.

## Results

### Siem Reap Clinic, 2004 and 2005

#### Patients

In September 2004, the Siem Reap chronic diseases clinic was actively following up on 1158 HIV positive people, 636 of which were not yet on ART and 522 of which were on ART. Among the 460 patients started on ART in 2004, the median CD4 count at initial assessment was 50 cells/μl (IQR: 20–117 cells/μl). The clinic was further following up on some 1000 patients with diabetes, hypertension and other chronic diseases. By August 2005, the clinic was following up on 1423 HIV positive people – 512 not yet on ART and 911 on ART – along with some 1700 patients with other chronic diseases. Among the 475 patients started on ART in 2005, median CD4 count at initial assessment was 75 cells/μl (IQR: 25–161 cells/μl).

#### Staff

In September 2004 the clinic had four full-time medical doctors doing the consultations for all chronic patients. There were two nurses, three counsellors, one pharmacist, one database operator and six PLWHAs – two involved in counselling, two in keeping files and guiding patients to the doctors, and two in organizing home visits of irregular and defaulter patients. In August 2005, the number of medical doctors was reduced to three full-time and one part-time. Other staff remained unchanged. The care delivered in the clinic can be described as doctor-based: every patient is seen by a doctor during every visit, and the doctor takes all decisions on diagnostic procedures and on treatment. The other staff members execute these decisions.

#### Consultations

The pre-ART phase for those patients with a CD4 count below 200 lasted on average 75 days, a time which did not vary much with the results of the CD4 counts. The clinic's database showed that during this preparation period the patients had on average 6 medical appointments until September 2004, and that this was reduced to 4 by August 2005. The time spent at each consultation remained constant at 15 minutes on average, and ranged between 8 and 23 minutes, depending on the patients' condition. In particular, patients with active opportunistic infections were often difficult to diagnose correctly with the limited technical means available and sometimes needed much more doctor-time before ART could be initiated.

A considerable number of total consultations are for people with a CD4 count above 200 (non-ART phase). These consultations lasted on average 15 minutes. As the total time patients stay in this phase is very variable, and can last several years, an estimation of the number of visits was impossible.

During ART initiation, the clinic doctors see the patient three times to prepare the patient for the initiation of ART. In 2004, these consultations lasted 30 minutes each. In 2005, the doctors had reduced the consultation time considerably to some 20 minutes per visit. We could indeed observe that non-medical counselling had been completely delegated to counsellors.

In September 2004 the clinic's database showed that during the first year of ART follow-up a patient had on average 14 consultations. Each visit lasted on average 12 minutes (ranging between 8 and 17 minutes). The length of the individual consultations did not change in August 2005, but the number of follow-up visits had reduced from 14 to 12.

In September 2004, there were only 139 patients in the second year of ART follow-up, most in the first six months. We counted seven consultations during these six months of the second follow-up year in the clinic's database. This suggested that the annual consultation rate of 14 visits would not change significantly after the first year of follow-up. Lacking a confirmed number, we assumed monthly (i.e. 12 annual) consultations as from the second year. No observation was made for the second year follow-up consultations, but the clinic doctors reckoned these to be slightly shorter than during the first year. For our calculations, we assumed an average length of 10 minutes per consultation. In August 2005 it was possible to calculate more accurately the actual frequency and length of consultations in the second year of follow-up. The average number was 10 consultations per patient. The average length of the individual consultation remained 10 minutes. These data are summarized in Table 1, and total doctor-time per patient is calculated.

**Table 1:** Medical doctor time for antiretroviral treatment (ART) in Siem Reap

	Number of consultations per patient		Minutes per consultation		Total doctor-time per patient in minutes		
	Sep-04	Aug-05	Sep-04	Aug-05	Sep-04	Aug-05	% change in 2005 compared to 2004

Pre-ART	6	4	15	15	90	60	- 33%
ART initiation	3	3	30	20	90	60	- 33%
ART follow-up year 1	14	12	12	12	168	144	- 14%
ART follow-up year 2	12	10	10	10	120	100	- 17%

Medical doctor-time for antiretroviral treatment (ART) for patients with initial CD4 < 200, September 2004 and August 2005, Chronic Diseases Clinic Siem Reap, Cambodia.

The total time needed for one PLWHA who went through the pre-ART phase, then the ART initiation, and who was followed up during year 1, was 348 minutes with the data observed in 2004, and 264 minutes with the 2005 data. Of this total reduction of 84 minutes, 54 minutes is due to a decrease in number of visits and 30 minutes due to a reduction in length of consultation. The result is a 24% reduction in doctor-time per patient in 2005, as compared to 2004.

In September 2004, the total number of consultations was 1433 for 1158 PLWHAs; 636 without ART and 522 on ART. In August 2005, there were 1492 consultations for 1423 PLWHAs; 512 without ART and 911 on ART. Table 2 presents how these consultations were divided between the different ART phases and how much doctor-time was needed for each phase.

**Table 2:** Total doctor time for consultation of HIV positive patients, Siem Reap

	Total doctor consultations		Minutes per doctor consultation		Total doctor consultation time in hours		
	Sep-04	Aug-05	Sep-04	Aug-05	Sep-04 (FTE)	Aug-05 (FTE)	Change 2005 compared to 2004

							
Pre-ART	276	176	15	15	69	44	-25 hours (-36%)
ART initiation	138	132	30	20	69	44	-25 hours (-36%)
ART follow up Year 1	462	484	12	12	92	97	+5 hours (+5%)
ART follow up Year 2	162	365	10	10	27	61	+34 hours (+126%)
Total ART	1038	1157			257 (2.06)	246 (1.97)	-11 hours (-4%)
							
Non-ART	395	335	15	15	99 (0.80)	84 (0.67)	-15 hours (-15%)
Total ART and non-ART	1433	1492			356 (2.86)	330 (2.64)	-26 hours (-7%)

Total doctor-time for consultation of HIV positive patients, September 2004 and August 2005, Chronic Diseases Clinic Siem Reap, Cambodia.

FTE = full-time equivalent (125 hours per month)

We thus estimated that in 2005 the doctors in the Siem Reap clinic spent 26 hours less on 1423 HIV positive patients than they spent in 2004 for 1158 patients (-7%). The most important gains are obtained in pre-ART and ART initiation, with 36% of reduction in time needed for these phases. This reveals a considerable gain in efficiency of use of doctors' time. There is a big increase (34 hours, or 126%) in the time spent on ART follow-up for the second and sub-sequent years, but this is more than compensated by time gains in earlier phases of ART.

### Siem Reap clinic simulation, 2004–2013

**Figure 2:**
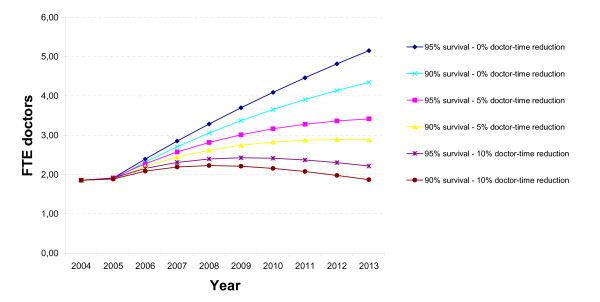
Need for doctors for ART in Siem Reap clinic, extrapolation 2004–2013.

Under the assumptions used, the total number of patients on ART grows to 480 at the end of 2004, to over 912 at the end of 2005, and to 3126 and 3852 at the end of 2013, for 90% and 95% annual survival respectively. The results for total doctor-time needed are shown in Figure 2. From 1.86 FTE doctor in 2004 and 1.89 FTE in 2005, the number of doctors required increases to more than 5 FTE for the scenario "95% survival – 0% doctor-time reduction", or to 4.35 FTE for the scenario "90% survival – 0% doctor-time reduction". Yet, it increases more moderately to around 3 FTE for the two scenarios with 5% annual doctor-time reduction, and increases only very slightly for the two scenarios with 10% annual doctor-time reduction.

### Extrapolation district-wide, 2004–2013

**Figure 3:**
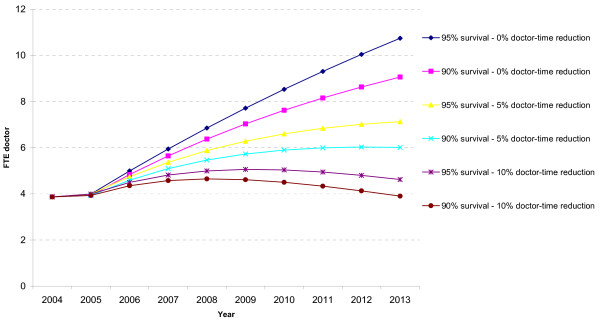
Need for doctors for ART in hypothetical district in sub-Saharan Africa, 2004–2013.

In line with assumptions for the sub-Saharan district (population 200,000; 20% adult HIV prevalence; 2000 patients yearly in need of ART; 50% coverage of ART needs), the number of patients on ART will gradually increase to 6 513 if survival is 90% per year, or to 8 026 if survival is 95% per year. The results for doctor time are presented in Figure 3. In all simulations, the initial need in 2004 for doctors is 3.87 FTE per year, and this increases to almost 11 FTE in 2013 in the scenario "95% survival – 0% doctor-time reduction", or to 3.90 FTE in the scenario "90% survival – 10% doctor-time reduction".

In the simulation for the hypothetical district in sub-Saharan Africa, the number of patients on ART per doctor increases to between 719 and 1736. The 'low estimate' of 719 ART patients per doctor is for the scenario "90% survival -0% doctor-time reduction". The 'high estimate' of 1736 ART patients per doctor is for the scenario "95% survival -10% doctor-time reduction".

## Discussion

Our observations in a chronic diseases clinic in Siem Reap confirm that ART is quite labour-intensive, with approximately 3 full-time doctors needed for treatment and follow-up of 1158 PLWHAs in September 2004. However, we documented important reductions in doctor-time per patient over one year with 24% less time needed in 2005 than in 2004 for putting one patient on ART and for follow-up over one year. These reductions in doctor-time were mainly due to a decrease in number of visits and less to a reduction in length of consultations. The most important gains were in the pre-ART and in ART initiation phases, with lesser gains in the ART follow-up phases. In the Siem Reap clinic, these reductions in doctor-time outweighed the increase in patient load. As a result, less doctor-time was needed for 1423 PLWHAs in August 2005 than for 1158 PLWHAs in September 2004. The doctors thought that these gains were possible mainly because of their own greater experience and better counselling by non-medical staff. It was striking that such steep reduction in doctor-time per patient occurred almost spontaneously. Doctors also thought that considerable further reductions in doctor-time per patient could be achieved, especially for patients in long-term follow-up, most of whom are stable and have few medical problems. Also, further reductions in the number of consultations were deemed possible. During our observations, all patients attending the clinic were seen by a doctor, while doctors agreed that stable uncomplicated patients could come for a refill and adherence counselling without need for medical consultation.

The simulation over 2004 – 2013 for Siem Reap clinic does not replicate exactly the evolution of the chronic disease clinic in Siem Reap documented over the period 2002 – 2005. We simulated that what actually happened between 2002 and 2005 took place over 2004 until 2005; thus with a start-up that was faster than what actually happened. However, with 480 patients on ART at the end of 2004 and 912 patients on ART at the end of 2005, the simulation comes quite close to the 522 patients on ART in September 2004 and 911 in August 2005. The extrapolation of these findings up to 2013, with a stable monthly influx of some 40 new PLWHAs, shows that the doctor-time needed is very sensitive to further reductions in doctor-time per patient, and to a lesser extent to annual survival rates of PLWHAs on ART.

The extrapolation of the doctor-based ART delivery model to the hypothetical district in sub-Saharan Africa (with 200,000 inhabitants and 20% adult HIV prevalence putting 50% of those in need on ART) over 2004 – 2013 shows that at least 4 full-time doctors would be needed for ART, and possibly as many as 11 full-time doctors under the most labour-intensive scenario.

Our data have serious limitations, especially the simulations. Estimating what is likely to happen in the future is difficult, certainly for something as new as large-scale life-long treatment in low-income countries, for which there is no precedent.

Inclusion of new patients in the Siem Reap clinic may not continue at the same rate of 40 new patients per month over a period of 10 years. Already in 2004 and 2005, the Siem Reap clinic was attracting growing numbers of PLWHAs from outside the province of Siem Reap. This may not continue, as the number of ART delivery sites has been growing rapidly in Cambodia. However, with an adult HIV prevalence of around 2% in its population of some 600 000, there were in 2004 an estimated 6000 adult PLWHAs in Siem Reap province, of which some 600 (10%) would need ART every year. So the 480 new inclusions per year in Siem Reap would cover 80% of the needs for ART for the province. To reach such coverage for a chronic disease for which demand for care is high may be realistic.

Estimations for annual survival of ART patients of between 90% and 95% are based on early experience from pilot projects with a high quality of care and very good adherence [1,2]. Whether such results can be maintained over a decade is uncertain. Recent experience from high-income countries shows that long-term annual survival on ART of 97% is possible [8,9], but this includes intensive laboratory monitoring and availability of second- and third-line treatments, which is as yet rarely the case in low-income countries. Data from a large-scale district-wide ART programme with simplified treatment schedules in Malawi revealed 76% survival after the first year, and 66% after two years [10]. Thus, our survival estimates may be too optimistic for large-scale ART provision in a rural district in sub-Saharan Africa.

Estimations for further reductions in doctor-time per patient include a wide range of assumptions, and seem to be realistic. With the present patient mix, further annual reductions of 10% in doctor-time needed seem realistic, thanks to doctors gaining more experience and more tasks being delegated to experienced nurses and counsellors. By 2013, patients on long-term follow-up would then receive on average 43 minutes of doctor-time per year. It seems quite realistic that by then such patients would be seen by doctors only 4 times per year, and for 10 minutes per consultation. However, at present only 1% of patients in the Siem Reap cohort are considered to be treatment failures and in need of second-line treatment. Such patients need considerably more doctor-time. The proportion of such 'difficult' patients will undoubtedly increase over time. Then the further reductions in doctor-time for 'routine' patients may be balanced out with the increased doctor-time needed for 'difficult' patients. Consequently, it is quite possible that 10% annual doctor-time reductions over a few more years are realized, but that doctor-time reductions would slow down, halt or even reverse in the longer-term. However, it may also be that the future brings further simplification in ART schedules or more robust combinations of anti-retroviral medicines with less need for monitoring and changing of regimens.

The assumptions for a district-wide ART scale-up may not be realistic, but the extrapolation is mainly intended to reveal the stakes in the domain of human resources. There are many districts in sub-Saharan Africa where adult HIV prevalence is stable around 20%. The natural evolution of HIV infection leads to death after approximately 10 years, and people are in need of ART one or two years before death. In a stable HIV epidemic, some 10% of all HIV positive adults will thus be newly in need of ART annually.

Whether covering 50% of ART needs is realistic depends mostly on funding, human resource constraints and organizational capacity. This varies widely between countries and within countries between regions and districts. We do not know whether enough funding will be made available over the long-term, but this does not seem at present the main bottleneck in many countries, thanks to the present commitments of the Global Fund, PEPFAR, the World Bank MAP and the State budgets. The present extrapolation shows clearly that the needs for doctors for such district-wide ART scale-up with a doctor-based ART delivery model are quite beyond their present availability, as the number of doctors in many health districts does not exceed 5, and is often far less; one or two doctors for an entire district with 200 000 inhabitants is not exceptional. So needing 4, 6 or even 11 doctors for ART is entirely beyond any reasonable possibility in most of the countries hardest hit by HIV/AIDS. Such countries may decide to increase intake in medical schools, or to import doctors from abroad. However, adopting ART delivery models that are nurse-based or centred around expert patients are other options that should be explored.

Particularly, the extrapolation of findings on use of doctor-time from Cambodia to sub-Saharan Africa may not be warranted. Indeed, there are important differences in medical practise and culture between countries and certainly between continents. However, the data published on ART from sub-Saharan Africa reveal that most ART clinics where doctor-time was documented used between 1 and 2 doctors per 1000 ART patients, which is quite similar to our findings from Siem Reap [5]. Only two clinics had considerably higher ratios, and many had far lower ratios. Moreover, anecdotal evidence shows that more far-reaching adaptations are being made on a piloted basis.

These differences between sites may be partly explained by a 'learning curve', well documented in a variety of medical techniques and procedures, but mainly used to explain better patient outcomes if procedures are performed by more experienced practitioners [11-14]. The learning curve is relatively steep in many medical procedures, with optimal results after one or two years of practise, and without further gains beyond that. However, the 'learning' in ART delivery is a more complex phenomenon of adapting a practise developed in resource-rich environments to low-income countries, while at the same time significantly scaling up. The learning is not only on the individual level of the providers of care, but also at the level of care teams, health care facilities, and support systems. These different layers of learning and adaptation can potentially have a multiplication effect, and may take more time for materialising. However, it is our contention that, more conscious efforts will have to be deployed to rationalize as far as possible the use of the precious time of qualified health workers, especially medical doctors.

## Conclusion

ART is labour intensive. Important reductions in doctor-time per patient can be realized during scaling-up. Estimations of the health workforce needs for ART [6] should take a dynamic perspective. Workforce planning based on the extrapolation of human resource use in pilot projects may ignore important doctor-time reductions that occur over time, even over relatively short time periods as during the one year we documented in Siem Reap.

Whether a doctor-based ART delivery model is feasible for scaling-up of ART depends largely on the context, and then mainly on the ratio of PLWHAs per doctor. The doctor-based ART delivery model analysed seems to be adequate for Cambodia. However, in many districts in sub-Saharan Africa such doctor-based ART delivery models may be incompatible with their HRH constraints.

ART is a rapidly globalizing lifesaving practise. However, given the current stocks of human resources for health, especially of doctors, practical ART delivery models should take into account the local human resource constraints and thus be context-specific.

To facilitate learning across sites and settings, it would be most useful if ART delivery sites did not only report their results in terms of patient outcomes, but also described the quantity and type of human resources for health they use, and to what extent they manage to delegate tasks to non-doctors, including medical assistants, nurse practitioners, lay providers and expert patients [15,16].

## Competing interests

The authors declare that they have no competing interests.

## Authors' contributions

Wim Van Damme designed the study, participated in the data collection, analysed the data, and wrote successive drafts of the manuscript. Katharina Kober participated in the design of the study, participated in data collection and data analysis, and reviewed successive drafts of the manuscript. Kheang Soy Ty participated in the data collection, provided the background information and reviewed successive drafts of the manuscript. Bart Janssens discussed the early results and reviewed successive drafts of the manuscript.
